# TRIM16: a context-dependent E3 ligase in autophagy, oxidative stress, and immune regulation – from cancer and systemic disease

**DOI:** 10.3389/fonc.2026.1743345

**Published:** 2026-02-04

**Authors:** Hanchen Dong, Jiayu Jiang, Ke Ni, Qinhong Ren, Xinran Yang, Liang Shang, Baoshan Cai, Leping Li

**Affiliations:** 1 Department of Gastroenterological Surgery, Shandong Provincial Hospital, Shandong University, Jinan, Shandong, China; 2 Department of Digestive Tumour Translational Medicine, Engineering Laboratory of Shandong Province, Shandong Provincial Hospital, Jinan, Shandong, China; 3 Department of Gastroenterological Surgery, Shandong Provincial Hospital of Shandong First Medical University, Jinan, Shandong, China; 4 Shandong Provincial Key Medical and Health Laboratory of Cell Metabolism, Central Hospital Affiliated to Shandong First Medical University, Jinan, Shandong, China; 5 Department of Clinical Medicine, Shandong First Medical University, Jinan, Shandong, China

**Keywords:** cancer, E3 ligase, lysophagy, oxidative stress, secretory autophagy, TRIM16

## Abstract

TRIM16 is an atypical tripartite motif (TRIM) family E3 ligase that retains catalytic activity via its RING-like B-box domains, functioning primarily as a versatile molecular scaffold. This architecture enables TRIM16 to coordinate key cellular processes. These include oxidative stress responses (via the p62-KEAP1-NRF2 axis), secretory autophagy, lysophagy, and immune regulation, which collectively maintain cellular homeostasis. Emerging evidence highlights TRIM16 as a context-dependent regulator whose functions are shaped by cell type and microenvironment. In cancer, it acts as a tumor suppressor in most solid tumors by degrading oncoproteins and inducing cell cycle arrest, yet can paradoxically promote progression in pancreatic and hepatocellular carcinomas. Beyond oncology, TRIM16 protects against neurodegenerative, cardiovascular, and inflammatory diseases through quality control mechanisms. This review integrates recent advances to elucidate TRIM16’s molecular mechanisms and disease-specific functions, emphasizing its therapeutic and biomarker potential, and outlines future directions to decode its context-dependent actions for clinical translation.

## 1 Introduction

In eukaryotic cells, the ubiquitin-proteasome system (UPS) serves as a central regulator of protein fate, with E3 ubiquitin ligases providing substrate specificity. The TRIM family represents a major class of E3 ligases, most of which contain a canonical RING domain that promotes protein degradation. This reflects the classical view of ubiquitin-mediated regulation. However, emerging evidence indicates that ubiquitination extends well beyond a “degradation tag,” playing critical non-degradative roles in signaling pathways such as autophagy, stress response, and immune regulation (1, 2).

TRIM16 exemplifies this functional diversification. As an atypical TRIM member lacking a RING domain but retaining E3 activity via its B-boxes, TRIM16 functions primarily as a molecular coordinator. This distinctive architecture enables it to orchestrate multiple cellular programs, including secretory autophagy, lysophagy, oxidative stress response, and immune signaling. Notably, TRIM16’s biological effects are highly context-dependent. It functions protectively in certain scenarios but contributes to pathology in others. This duality is especially apparent in cancer. This review synthesizes recent advances by first delineating the core cellular roles of TRIM16 as a homeostatic integrator. We then examine how disruptions or hijacking of these functions drive its diverse contributions to human disease. Finally, we discuss its translational potential as a therapeutic target and biomarker, and outline future directions for harnessing its biological complexity to inform clinical applications.

## 2 The unique architecture of TRIM16: an atypical E3 ligase

TRIM16 stands out within the large TRIM family of E3 ubiquitin ligases due to its atypical domain composition and its embodiment of non-canonical ubiquitin-related functions.

### 2.1 Domain organization and catalytic mechanism

The Tripartite Motif (TRIM) family represents a group of evolutionarily conserved proteins characterized by a tripartite domain architecture composed of a RING finger, one or two B-box domains, and a coiled-coil region. To date, more than 80 TRIM proteins have been identified (3). They participate in a broad range of biological processes, including apoptosis, cell cycle control, antiviral defense, and ubiquitin-dependent signaling. Functionally, most TRIM proteins act as E3 ubiquitin ligases, bridging E2 ubiquitin-conjugating enzymes via the RING domain and recognizing specific substrates through their B-box or coiled-coil domains, thereby catalyzing ubiquitin transfer to target proteins (4). TRIM16 (previously known as estrogen-inducible protein 117 EBPP) is one of the more ancient members of the TRIM family and was initially identified in relation to estrogen response (5). The TRIM16 gene is located on chromosome 17p11.2, and the encoded protein is primarily cytoplasmic, with a minor nuclear fraction (6). In neuroblastoma cells and normal keratinocytes, nuclear localization of TRIM16 is enhanced upon retinoic acid stimulation (7, 8).

**Figure 1:**
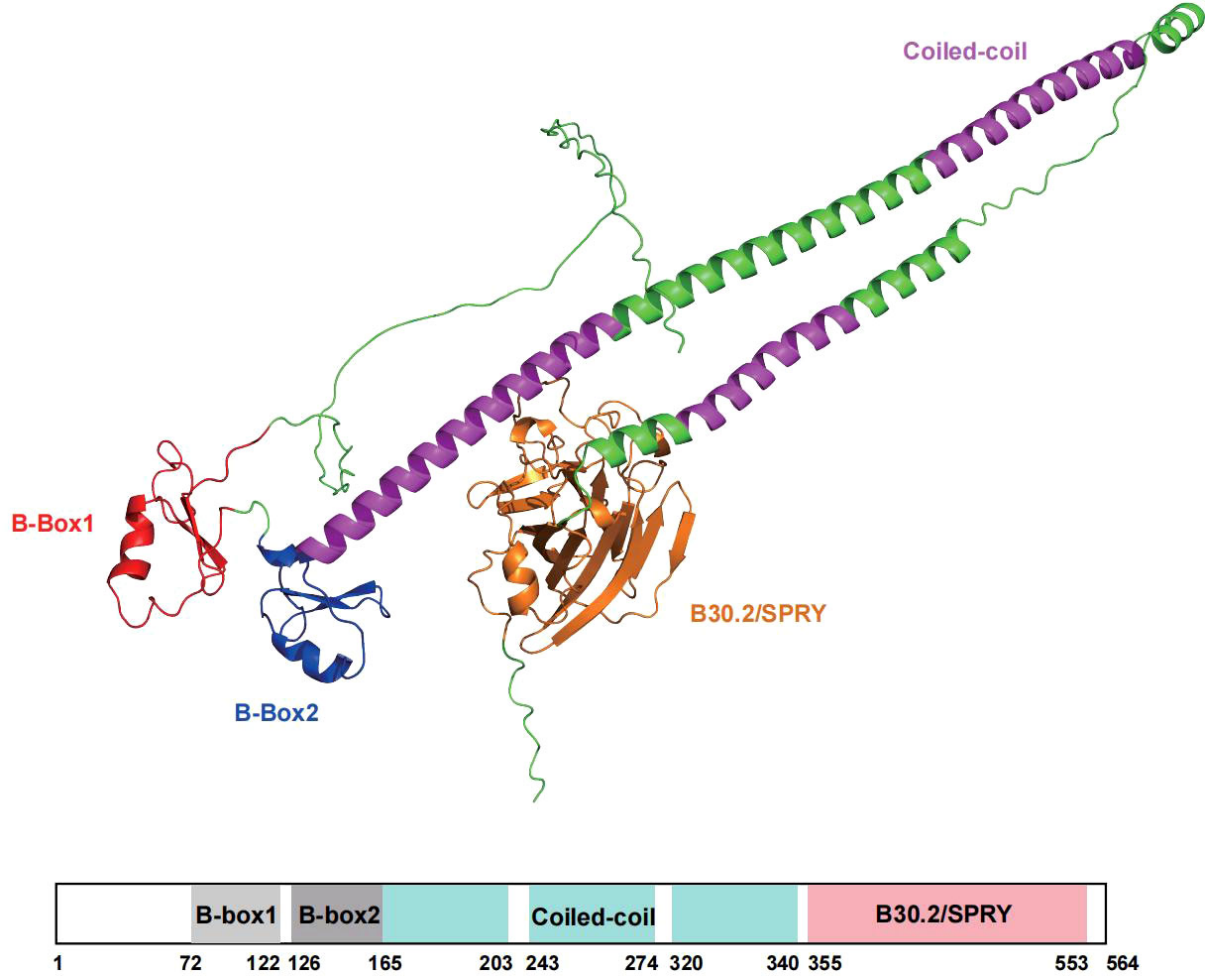
The spatial structural framework and domains of the TRIM16 protein. This figure presents the domain architecture of the TRIM16 protein. The protein is depicted in a schematic form, highlighting its various functional domains. The N-terminal region contains the B-Box1 domain and the B-Box2 domain. The B-box domains adopt a RING-like fold, enabling the TRIM16 protein to have E3 ligase activity. The middle part of the protein has a coiled-coil region, which is crucial for protein dimerization and interaction with other molecules. The C-terminal region contains an SPRY domain, which is the core of its inflammatory regulation function and can directly bind to the C-termini of proIL-1β and mature IL-1β. The bottom of the figure also includes a scale bar, indicating the amino acid positions along the protein sequence. The protein structure was predicted using AlphaFold3 and visualized using Pymol.

Structurally, TRIM16 contains two B-box domains (B-box1 and B-box2), a coiled-coil region, and a C-terminal SPRY (B30.2) domain (**Figure 1**). Although lacking the canonical RING domain, its B-boxes adopt a RING-like fold that confers E3 ligase activity. This unique configuration may represent an ancestral prototype of early TRIM proteins. It suggests that during evolution, some TRIM members diverged structurally: they lost the RING domain while enhancing the catalytic potential of their B-box motifs. TRIM16 can form homodimers through its coiled-coil region and also heterodimerize with other TRIM proteins, including TRIM18 and TRIM19 (9).The C-terminal SPRY domain of TRIM16, composed of approximately 200 amino acids, adopts a β-sheet-rich globular structure that plays a key role in inflammation regulation. This domain directly binds both pro-IL-1β and the C-terminal segment of mature IL-1β, promoting their unconventional secretion through the autophagic pathway. Moreover, TRIM16 interacts with inflammasome components such as the p20 and p10 subunits of Caspase-1 and the NACHT domain of NALP1, facilitating the assembly of a functional inflammasome complex and amplifying inflammatory signaling (10).

### 2.2 Substrate recognition: insights from known interactors

Current evidence indicates that TRIM16 substrates lack a single, conserved linear amino acid motif. Recognition is instead achieved through domain-specific interactions between TRIM16 and structural epitopes on its binding partners. The B-box domains mediate binding to the head domain of Vimentin (7, 8, 11) and are required for interaction with the transcription factor Snail (12). The C-terminal SPRY domain directly engages the C-terminal segment of mature IL-1β (10, 13, 14) and also binds the autophagy adaptor p62 (15). Additionally, dimerization via the coiled-coil region can modulate these interaction interfaces (9). This domain-centric recognition strategy forms the structural basis for TRIM16’s selective partnership with diverse proteins.

## 3 Core cellular functions of TRIM16: from homeostasis to stress adaptation

### 3.1 TRIM16 and basal homeostasis

TRIM16 serves pivotal and multifaceted roles in maintaining cellular and tissue homeostasis. Under physiological conditions, it functions as a protective molecular scaffold that orchestrates several fundamental homeostatic mechanisms, including redox balance and protein quality control through selective autophagy and lysophagy. As a central coordinator of cellular quality control under basal conditions, TRIM16 continuously fine-tunes the cellular antioxidant response. Through its scaffolding function, it helps maintain redox balance by protecting cells from endogenous reactive oxygen species (ROS) (15). This basal regulatory activity is essential for preventing oxidative damage during routine cellular metabolism.

Concurrently, TRIM16 participates in constitutive quality control mechanisms that extend beyond stress-induced responses. Its involvement in selective autophagy and lysophagy represents a fundamental cellular housekeeping system for clearing damaged organelles and protein aggregates, thereby preserving proteostasis even in the absence of external stressors (13, 14).These dual functions establish TRIM16 as a key guardian of cellular homeostasis. This foundational role has important implications for understanding how TRIM16 dysregulation may contribute to disease initiation.

### 3.2 TRIM16 and cell fate regulation

Beyond its roles in general cellular maintenance, TRIM16 directly governs critical cell fate decisions by regulating both proliferation and differentiation programs. This regulatory capacity is essential for normal development, tissue integrity, and physiological renewal. TRIM16 exerts precise control over cell cycle progression, particularly at the G1/S transition checkpoint. This control is achieved through multiple complementary mechanisms. These include stabilizing TDP43, reducing phosphorylation of E2F1 and pRb, and modulating key cell cycle proteins such as cyclin D1 and p27 (7, 8, 16–18).Through these coordinated actions, TRIM16 enforces the G1/S checkpoint, ensuring orderly cell cycle progression.

In parallel, TRIM16 coordinates tissue-specific differentiation programs in various contexts. In the epidermis, it participates in keratinocyte terminal differentiation, a process vital for maintaining skin barrier function (6). During osteogenic differentiation of human bone marrow mesenchymal stem cells (hBMSCs), TRIM16 acts as a positive regulator through autophagy-dependent mechanisms. It forms a functional complex with Galectin-3, ULK1, and Beclin1 to enhance autophagic activity, while simultaneously promoting RUNX2 stability by targeting the E3 ligase CHIP for degradation (19, 20).These functions enable TRIM16 to coordinate proliferation and differentiation, maintaining tissue homeostasis. Their dysregulation contributes to disease.

### 3.3 TRIM16 and oxidative stress amplification

TRIM16 serves as a critical amplifier within the cellular antioxidant defense system. Under oxidative or proteotoxic stress, it transitions from a basal maintenance factor to a potent enhancer of the p62-KEAP1-NRF2 pathway. Mechanistically, TRIM16 coordinates a multi-step amplification cascade. It facilitates the autophagic degradation of the repressor KEAP1 by promoting its sequestration via p62 (15).This interaction liberates the transcription factor NRF2, whose basal regulation involves KEAP1-mediated ubiquitination and degradation (21). TRIM16 further enhances NRF2 activity by modulating its ubiquitination. It reduces K48-linked chains while promoting K63-linked modifications, thereby stabilizing NRF2 and promoting its nuclear translocation. Activated NRF2 then drives the expression of antioxidant genes (e.g., HMOX1, NQO1) and creates a self-reinforcing positive feedback loop by upregulating p62 and TRIM16 expression (15, 21).

TRIM16 demonstrates protective functions across diverse oxidative stress models. In esophageal squamous cell carcinoma cells, its expression is markedly induced by Oridonin, mirroring the response to glutathione depletion and suggesting an adaptive antioxidant role (22). In lung epithelial cells exposed to urban particulate matter, TRIM16 expression correlates with ferroptotic markers like ROS accumulation and lipid peroxidation, indicating involvement in oxidative-iron metabolic crosstalk (23).In Baikal whitefish, TRIM16 cooperates with heat shock proteins (HSP30–HSP90) to maintain proteostasis under thermal stress (24).Thus, by scaffolding the p62-KEAP1-NRF2 axis and establishing a positive feedback loop, TRIM16 acts as a decisive amplifier of antioxidant signaling. This capacity for context-dependent signal potentiation is central to its function in stress adaptation.

### 3.4 TRIM16 and autophagic trafficking

TRIM16 plays a pivotal role in specialized membrane trafficking pathways, functioning as both a cargo receptor for unconventional secretion and a quality control scaffold for damaged organelles. In secretory autophagy, TRIM16 acts as a key receptor that facilitates the unconventional secretion of specific proteins. It directly binds mature IL-1β via its SPRY domain and packages it into autophagosomes (14). This process involves interactions with the R-SNARE Sec22b for cargo recruitment to LC3-II positive membranes, followed by cooperation with plasma membrane SNAREs (Syntaxin 3/4, SNAP-23/29) to enable autophagosome-plasma membrane fusion and extracellular release, bypassing lysosomal degradation. Galectin-8 further contributes by interacting with TRIM16 (13, 14). Beyond IL-1β, TRIM16 mediates ferritin secretion through the TRIM16-Sec22b pathway, though cell-type specificity exists as astrocytes utilize TRPML1-mediated lysosomal exocytosis instead (13, 25).

**Figure 2:**
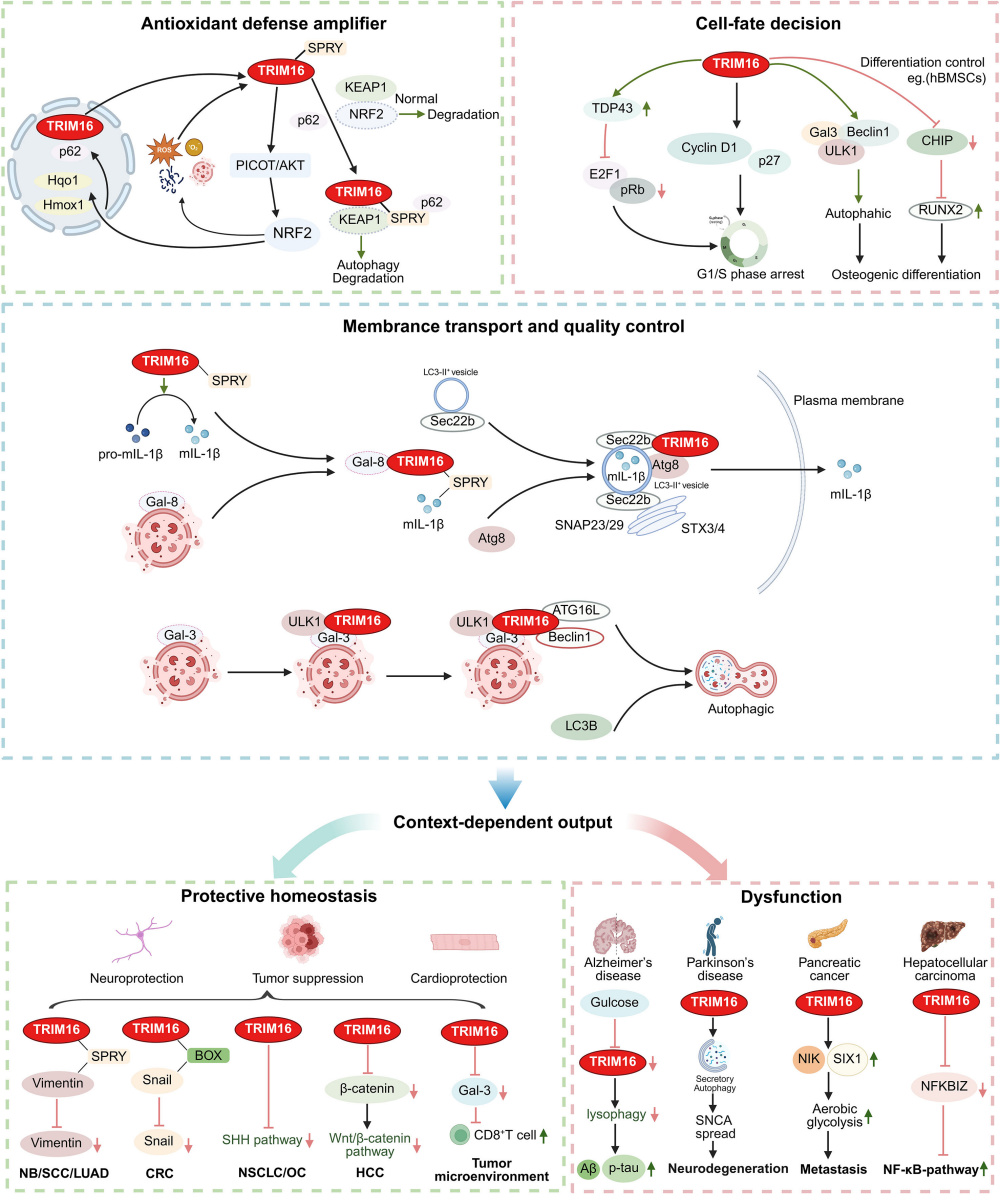
Multi-level regulatory network controlling TRIM16 expression. This figure illustrates the transcriptional, post-transcriptional, and post-translational mechanisms that regulate TRIM16 expression in various diseases. Transcriptional control: In NASH, EGR2 activates TRIM16 transcription. In ovarian cancer, the CRABP2/EZH2/DNMT1 complex modulates TRIM16 promoter activity. Post-transcriptional control: Multiple non-coding RNAs and RNA-binding proteins regulate TRIM16 mRNA stability and availability, including lncRNA OTUD6B-AS1, lncRNA CASC2, circPTK2, HuR, miR-214, and miR-942.Post-translational control: In ESCC, RASSF6 influences TRIM16 protein levels, while in glioblastoma, EPRL2 modulates TRIM16 stability via the ERK1/2 signaling pathway. Created in https://BioRender.com.

Concurrently, TRIM16 is essential for lysophagy, the selective autophagy of damaged lysosomes. Upon lysosomal damage, Galectin-3 initially recruits ESCRT components for membrane repair. When repair fails, TRIM16 is recruited to damage sites via Galectin-3 in a ULK1-dependent manner, where it scaffolds ATG16L1, LC3B, and Beclin1 to eliminate irreparably damaged lysosomes (15, 26, 27). This protects cells from the oxidative and proteotoxic stress caused by lysosomal content leakage. Through these distinct but complementary mechanisms, TRIM16 orchestrates crucial membrane trafficking events: directing specific proteins for unconventional secretion while ensuring the removal of dysfunctional organelles, thereby maintaining cellular integrity under diverse conditions (**Figure 2**).

### 3.5 TRIM16 and immune regulation

TRIM16 serves as an important regulator at the intersection of cellular stress responses and immune signaling, modulating both inflammatory pathways and antiviral defense mechanisms. In inflammasome regulation, TRIM16 helps prevent excessive inflammatory activation. Its expression is significantly reduced in the periodontal tissues of patients with periodontitis compared to healthy individuals, suggesting a protective role in inflammatory control. Mechanistically, TRIM16 binds proIL-1β and interacts with inflammasome components including pro-caspase-1 and NLRP1, thereby enhancing IL-1β production while potentially maintaining regulatory balance (28). Under H_2_O_2_-induced oxidative stress in human periodontal ligament stem cells (hPDLSCs), TRIM16 levels decrease in a dose- and time-dependent manner. Overexpression of TRIM16 alleviates oxidative damage by activating the PICOT/Akt/Nrf2 signaling pathway, reducing ROS and related oxidants, and restoring osteogenic differentiation inhibited by H_2_O_2_ (29).

In antiviral defense, TRIM16 exhibits cell-type specific antiviral activity. In HEK293T cells, TRIM16 overexpression inhibits replication of multiple viruses (RSV, IAV, HSV-1, HMPV, PIV-3) and reduces viral protein synthesis, though this effect is not observed in A549, HeLa, Hep2, or Vero cells, highlighting its cell-context dependent nature (30). In A549 cells during H5N1 infection, TRIM16 regulates the p62-NRF2-KEAP1 axis to balance oxidative stress, lowering viral titers while upregulating antioxidant genes (GCLC, GCLM, HO-1, NADPH, GPX2) (31).Additionally, in NHDF cells infected with porcine endogenous retrovirus (PERV), TRIM16 expression increases, suggesting its function as a restriction factor in innate antiviral immunity (32).

In antitumor immunity, TRIM16 expression is upregulated in immune responders among hepatocellular carcinoma patients three weeks after atezolizumab-bevacizumab treatment, indicating its potential as a biomarker for immunotherapy response (33). Through these diverse mechanisms, TRIM16 maintains immune homeostasis by fine-tuning inflammatory responses and contributing to cell-intrinsic antiviral defense.

## 4 Regulation of TRIM16 expression and activity

**Figure 3:**
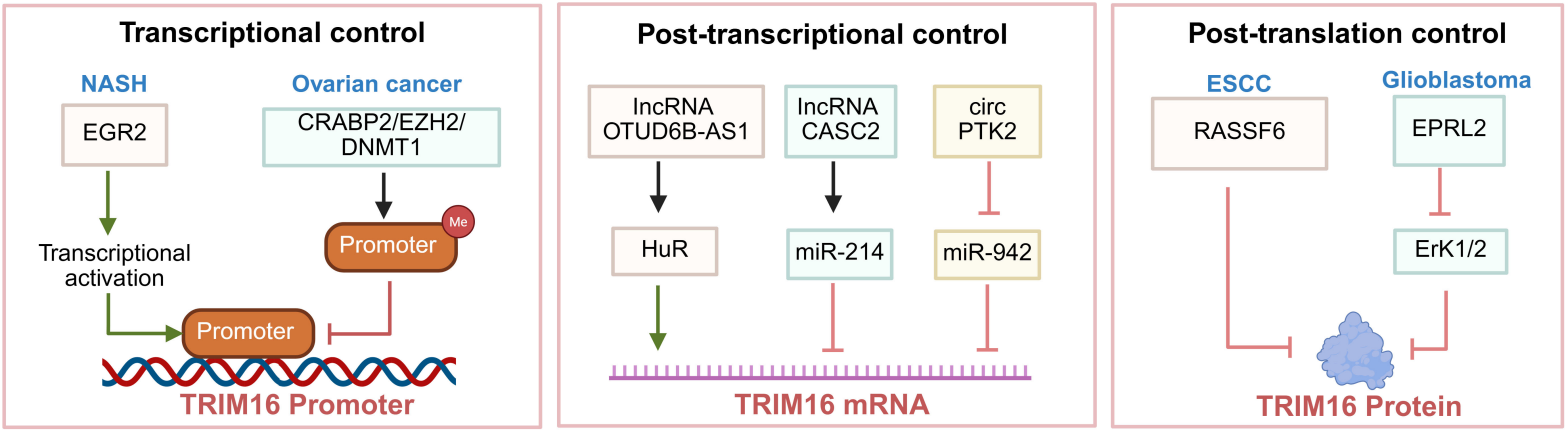
Functional roles of TRIM16 in cellular processes. This figure outlines the multifunctional roles of TRIM16 in cellular regulation. It illustrates how TRIM16 acts as an antioxidant defense amplifier, influencing autophagy and degradation processes. Additionally, it shows TRIM16’s involvement in cell-fate decisions, including differentiation control and autophagy regulation. The figure also depicts TRIM16’s role in membrane transport and quality control, affecting processes like IL-1β maturation and autophagy initiation. Lastly, it highlights the context-dependent outputs of TRIM16, which can either support protective homeostasis or contribute to disease pathogenesis across various conditions such as neurodegeneration and cancer. Created in https://BioRender.com.

The expression and function of TRIM16 are under sophisticated multi-layer control, encompassing transcriptional, post-transcriptional, and post-translational regulation. This intricate regulatory network is fundamental to its context-specific actions and contributes significantly to its dual roles in physiology and disease (**Figure 3**).

### 4.1 Transcriptional regulation

TRIM16 transcription is modulated by specific transcription factors and epigenetic mechanisms. In ovarian cancer, CRABP2 induces TRIM16 promoter methylation via EZH2/DNMT1, leading to its transcriptional repression and facilitating epithelial–mesenchymal transition(EMT) (34). Furthermore, in non-alcoholic steatohepatitis (NASH), the transcription factor EGR2 directly activates TRIM16 transcription, highlighting a stress-responsive transcriptional upregulation (35).

### 4.2 Post-transcriptional regulation

A complex network of non-coding RNAs fine-tunes TRIM16 mRNA stability and translation. MicroRNAs (miRNAs) are prominent negative regulators. For instance, miR-942 directly targets TRIM16 mRNA, contributing to cisplatin resistance in non-small cell lung cancer (NSCLC). Competing endogenous RNA (ceRNA) networks provide a counterbalancing mechanism. The circular RNA circPTK2 sponges miR-942, thereby alleviating its repression on TRIM16 and restoring chemosensitivity in NSCLC (36).Long non-coding RNAs (lncRNAs) also play pivotal roles: lncRNA CASC2 acts as a sponge for miR-214, indirectly inhibiting TRIM16 mRNA in CRC, while lncRNA OTUD6B-AS1 stabilizes TRIM16 mRNA by recruiting the RNA-binding protein HuR, promoting ferroptosis and radiosensitivity (37, 38).

### 4.3 Post-translational regulation and stability control

TRIM16 protein stability and activity are themselves regulated by ubiquitination and interactions within specific pathways. The protein can be targeted for proteasomal degradation. In esophageal squamous cell carcinoma (ESCC), RASSF6 promotes the ubiquitination and subsequent degradation of TRIM16, enhancing tumor aggressiveness (39). Furthermore, its functional output is modulated by upstream signaling nodes. For example, in glioblastoma, NPRL2 increases TRIM16 expression via inactivation of ERK1/2, illustrating how TRIM16 can be directed by upstream regulators within a specific pathway (40).

## 5 TRIM16 in human diseases: context-dependent dysregulation of core functions

### 5.1 Cancer

TRIM16 plays a strikingly dual role in cancer. While it acts primarily as a tumor suppressor in most solid tumors, it paradoxically promotes tumor progression in specific cancer types. Its involvement is underscored by genetic and epigenetic alterations across tumor types, including genetic variants and promoter hypermethylation, which collectively influence its expression and function within the cancer genetic landscape.

#### 5.1.1 Genetic and epigenetic alterations of TRIM16 in cancer

The involvement of TRIM16 in cancer genetics is underscored by specific genetic and epigenetic alterations identified across tumor types. In hepatocellular carcinoma, a specific genetic variation, TRIM16 E121D, has been identified. This variant corresponds to the missense mutation rs2074890 (G > T), which results in the substitution of glutamic acid with aspartic acid at amino acid position 121 (E121D). This genetic alteration has been associated with both the risk of developing HCC and its prognosis. Specifically, individuals carrying the rs2074890 T allele (encoding TRIM16 E121D) exhibit a significantly reduced risk of HCC and improved overall survival compared to those homozygous for the rs2074890 G allele (encoding TRIM16 E121E). Mechanistically, TRIM16 E121D displays a markedly enhanced ability to inhibit the proliferation, migration, and invasion of HCC cells, thereby contributing to the protective effects observed against HCC development and progression (41). In ovarian cancer, the CRABP2/EZH2/DNMT1 axis promotes promoter methylation and transcriptional repression of TRIM16, facilitating EMT (34). There are no clear reports regarding the somatic variations and copy number changes of TRIM16. These findings exemplify how TRIM16 is integrated into the genetic and epigenetic landscape of cancer, influencing not only cellular phenotypes but also disease susceptibility and progression at the population and individual tumor level.

#### 5.1.2 Tumor-suppressive mechanisms

In numerous cancers, TRIM16 acts as a potent tumor suppressor through multiple mechanisms:

##### 5.1.2.1 Inhibition of metastasis and EMT

In neuroblastoma, cutaneous squamous cell carcinoma, and lung adenocarcinoma, TRIM16 binds the Head domain of Vimentin via its C-terminal SPRY domain and mediates its K48-linked polyubiquitination and degradation, thereby inhibiting cytoskeletal dynamics essential for metastasis (7, 8, 11). In colorectal cancer (CRC), it directly targets the transcription factor Snail through its B-box domain for degradation, suppressing EMT and metastatic potential (12). Similarly, in NSCLC and ovarian cancer, low TRIM16 levels facilitate EMT via the sonic hedgehog (SHH) pathway (42, 43). TRIM16 suppresses melanoma proliferation and migration through IFNβ1, and its stability is enhanced by BRAF inhibitor vemurafenib or Withaferin A, suggesting potential combinatorial therapies (44–46).

##### 5.1.2.2 Suppression of oncogenic signaling

Beyond cytoskeletal and transcriptional regulators, TRIM16 also degrades central oncogenic signaling molecules. In hepatocellular carcinoma, it promotes the degradation of β-catenin, thereby inhibiting the Wnt/β-catenin signaling axis and the expression of downstream oncogenes like c-MYC and Cyclin D1 (41).

##### 5.1.2.3 Induction of apoptosis and cell cycle arrest

TRIM16 overexpression induces apoptosis through activation of caspase-2 in cancer cells (47). TRIM16 stabilizes TDP43, downregulates E2F1 and pRb phosphorylation, and induces G1/S arrest in neuroblastoma, breast cancer, and cutaneous carcinoma (7, 8, 16). In neuroblastoma, TRIM16 peaks in G1, localizes to the nucleus, and delays the G1/S transition via Cyclin D1 and p27 (17). Retinoic acid (RA) enhances differentiation and apoptosis, in RA–resistant cancer cells, TRIM16 suppresses proliferation by reducing Cyclin D1 and pRb phosphorylation, and it promotes RA response by activating RARβ2 transcription and acetylating histone H3, restoring RA sensitivity in resistant cells (18, 48).

##### 5.1.2.4 Reversal of therapy resistance

TRIM16 plays a crucial role in overcoming drug resistance. Its downregulation by miR-942 contributes to cisplatin resistance in NSCLC, an effect reversed by the circRNA circPTK2 which sponges miR-942 and restores TRIM16 expression (36). In glioblastoma, compounds like sanggenol L upregulate TRIM16, which then promotes the degradation of OPTN, inhibits protective mitophagy, and sensitizes cells to temozolomide (49). In CRC, stabilization of TRIM16 mRNA by lncRNA OTUD6B-AS1 enhances GPX4-mediated ferroptosis and radiosensitivity (38).

#### 5.1.3 Tumor promotion mechanisms

In contrast to its predominant tumor-suppressive function, TRIM16 can exhibit oncogenic properties in specific cancers, primarily by stabilizing pro-tumorigenic factors or activating survival pathways.

##### 5.1.3.1 Promotion of tumor progression

High TRIM16 expression correlates with vascular invasion, lymph node metastasis, and poor prognosis in pancreatic cancer. Mechanistically, TRIM16 stabilizes the kinase NIK and the transcription factor SIX1 in a ligase-independent manner, enhancing aerobic glycolysis and driving metastasis (50). This oncogenic role makes TRIM16 a potential target for inhibition, as demonstrated by delphinidin which suppresses TRIM16 and inhibits the PI3K/AKT pathway in a p53-dependent manner (51).

##### 5.1.3.2 Mediation of therapy resistance

In HCC, TRIM16 exerts an oncogenic function by promoting the degradation of NFKBIZ, a negative regulator of NF-κB. This leads to constitutive activation of NF-κB signaling, upregulation of anti-apoptotic proteins like BCL-2 and Survivin, inhibition of Caspase-3/Bax, and consequently, enhanced resistance to the targeted therapy sorafenib (52).

#### 5.1.4 Modulating the tumor immune microenvironment

Beyond direct effects on cancer cells, TRIM16 influences anti-tumor immunity. In glioma, under the regulation of NPRL2, TRIM16 promotes the degradation of the Galectin-3. This action helps restore the cytotoxic function of CD8^+^ T cells and alleviates the immunosuppressive tumor microenvironment (40, 53). Furthermore, in HBV-related HCC, TRIM16 expression correlates with tumor mutation burden and immune infiltration, suggesting a role in shaping the immunogenic landscape of the tumor (54, 55).

In summary, TRIM16’s role in cancer is profoundly context-dependent. It primarily functions as a tumor suppressor by degrading oncoproteins, arresting the cell cycle, and sensitizing cells to therapy. However, in specific settings like pancreatic cancer and a subset of HCC, it adopts an oncogenic role by stabilizing pro-growth factors or activating resistance pathways. This duality necessitates a precise understanding of the molecular context when considering TRIM16 as a therapeutic target.

### 5.2 Neurodegenerative diseases

TRIM16 is implicated in neurodegenerative disorders primarily through its regulation of autophagy and lysosomal integrity, processes critical for clearing toxic protein aggregates. In Parkinson’s disease, TRIM16-mediated secretory autophagy may paradoxically contribute to pathology by promoting the intercellular spread of α-synuclein(SNCA), thereby exacerbating neurodegeneration (56).In diabetes-associated Alzheimer’s disease (AD), TRIM16 expression is reduced in high glucose-treated human iPSC-derived neurons, mouse hippocampal neurons, and hippocampi of STZ-induced diabetic mice. mTORC1 inhibits TFEB, downregulating TRIM16, impairing lysophagy, and increasing lysosomal membrane permeability. This disrupts degradation of amyloid-β (Aβ) and phosphorylated tau (p-MAPT). TRIM16 overexpression restores lysophagy, recruits LC3, p62, and ubiquitin to damaged lysosomes, enhances lysosomal enzyme activity (CTSB, CTSD), reduces Aβ and p-MAPT accumulation, and improves cognitive function (57).In age-related macular degeneration, TRIM16’s role in secretory autophagy again comes to the fore, making it a potential therapeutic target. Inhibiting TRIM16 could selectively block the harmful secretion of neurotoxic factors like Aβ and lipofuscin without compromising core degradative autophagy (58).

### 5.3 Cardiovascular and cerebrovascular diseases

TRIM16 provides comprehensive protection against cardiovascular and cerebrovascular injuries through ubiquitination-dependent regulation of oxidative stress, inflammation, and cell death pathways. In doxorubicin-induced cardiotoxicity, TRIM16 is upregulated in cardiomyocytes and mediates the ubiquitination and degradation of p-TAK1, inhibiting JNK/p38 MAPK signaling, reducing inflammatory cytokine release (TNF-α, IL-6), and attenuating apoptosis and fibrosis. Additionally, it promotes YAP nuclear translocation and activates the Nrf2 antioxidant pathway, lowering oxidative damage markers (MDA, LDH, CK-MB) and enhancing antioxidant enzymes (SOD, CAT, GSH).TRIM16 overexpression improves cardiac function in pressure overload and pathological hypertrophy models (59).TRIM16 is highly expressed in human heart failure and mouse hypertrophy models. Cardiomyocyte-specific knockout of TRIM16 worsens hypertrophy and fibrosis. Mechanistically, it mediates Src ubiquitination, enhancing Prdx1 activity and Nrf2 signaling, upregulating antioxidant genes (HO-1), reducing ROS, and suppressing hypertrophy (60). In chronic kidney disease models, TRIM16 reduces cardiomyocyte hypertrophy via RIP2 degradation and p38 inhibition. Its downregulation by indole-3-acetic acid can be reversed by Saikosaponin A, improving diastolic function and hypertrophy (61).

Against myocardial ischemia/reperfusion injury, TRIM16 acts via multiple mechanisms: it reduces ROS, improves cell survival, decreases infarct size, and lowers serum markers through the Keap1/Nrf2 pathway (62), and separately suppresses inflammatory cytokines by promoting NLRP3 ubiquitination and degradation, inhibiting inflammasome assembly and pyroptosis (63).In cerebral stroke models, TRIM16 expression is induced in hippocampal neurons and protects by downregulating KEAP1 and activating the NRF2/ARE pathway, reducing ROS and neuronal apoptosis (64).In summary, TRIM16 exerts protective effects in cardiovascular and cerebrovascular diseases via ubiquitination, antioxidant defense, anti-inflammatory responses, and anti-apoptotic mechanisms, highlighting its broad potential as a therapeutic target.

### 5.4 Other diseases

TRIM16 exerts protective roles in various non-neoplastic diseases by regulating autophagy, ubiquitination-mediated degradation, and antioxidant pathways, thereby maintaining tissue homeostasis and mitigating cellular stress. In chronic obstructive pulmonary disease (COPD), TRIM16 positively correlates with FEV1.0%, while Galectin-3 correlates with smoking index but inversely with FEV1.0%. In patient HBECs, TRIM16 is reduced, whereas LAMP1 and Galectin-3 are elevated. Cigarette smoke extract triggers lysosomal membrane permeabilization (LMP), and TRIM16, together with Galectin-3, mediates lysophagy to preserve lysosomal function. Loss of TRIM16 increases lipofuscin, senescence proteins, and cellular aging, indicating that impaired lysophagy contributes to COPD pathogenesis (65).

In NASH, TRIM16 is transcriptionally activated by EGR2 and upregulated in hepatocyte lipotoxicity models as well as in livers from NASH patients and HFD/HFHC-fed mice. TRIM16 overexpression reduces lipid accumulation and inflammatory factor expression by mediating K48-linked ubiquitination and degradation of p-TAK1 via its SPRY/B30.2 domain, thereby inhibiting TAK1-JNK/p38 signaling, downregulating lipogenesis genes (Fasn, Acca), and upregulating fatty acid oxidation genes (Ppara, Cpt1a) (35).In age-related sarcopenia, TRIM16 alleviates D-galactose-induced oxidative stress by activating the PI3K/AKT/mTOR pathway via SIRT1, inhibiting FOXO3, upregulating antioxidant proteins, increasing myogenic markers (MHC, MyoD, MyoG), and suppressing E3 ubiquitin ligases (Atrogin-1, MuRF-1), thereby mitigating myotube atrophy (66). In senile osteoporosis (SOP), TRIM16 is downregulated in aging models and senescent osteoblasts. It promotes osteogenic differentiation via two mechanisms: activating the p62-KEAP1-NRF2 antioxidant pathway and stabilizing the transcription factor Runx2 to enhance ALP activity and mineralization (**Table 1**) (67).

**Table 1:** The role of TRIM16 in diseases and its molecular mechanism.

Disease area		Major diseases	TRIM16 expression/Function	Molecular mechanism	References
Cancer	Anti-carcinogenic effect	Neuroblastoma, cutaneous squamous cell carcinoma, lung adenocarcinoma, colorectal cancer	Inhibits EMT and metastasis potential.	Promotes degradation of proteins like Vimentin, Snail.	(7, 8, 11, 12)
		Hepatocellular carcinoma, non-small cell lung cancer, ovarian cancer	Tumor suppressor.	Inhibits Wnt/β-catenin and SHH pathways.	(41–43)
		Melanoma	Therapeutic targets of vemurafenib or Withaferin A.	Inhibits tumors by blocking IFNβ1, and its stability can be enhanced by the BRAF inhibitors.	(44–46)
		Neuroblastoma, cutaneous carcinoma, breast cancer	Tumor suppressor.	Stabilizes TDP43, downregulates E2F1 and pRb, induces G1/S arrest.	(7, 8, 16)
		Non-small cell lung cancer	Reverses the chemoresistance to cisplatin.	CircPTK2 reversed the miR-942-induced downregulation of TRIM16.	(36)
		Glioblastoma	Sensitizes cells to temozolomide.	Sanggenol L upregulates the TRIM16, promoting the degradation of OPTN, inhibiting protective mitophagy.	(49)
		Colorectal cancer	Enhances GPX4-mediated ferroptosis and radiosensitivity.	Stabilization of TRIM16 mRNA by lncRNA OTUD6B-AS1.	(38)
	Carcinogenic effect	Pancreatic cancer	Promotes aerobic glycolysis and tumor metastasis.	Stabilizes NIK and SIX1.	(50)
		Hepatocellular carcinoma	Enhance resistance to sorafenib.	Degrades NFKBIZ, activating NF-κB pathway.	(52)
	Tumor microenvironment	Glioma	Alleviates the immunosuppressive tumor microenvironment.	Regulated by upstream NPRL2, promoting Galectin-3 degradation, ameliorating CD8+ T cell exhaustion.	(40, 53)
		HBV-related HCC	Shapes the immunogenic landscape of the tumor.	Correlates with tumor mutation burden and immune infiltration.	(54, 55)
Neurodegenerative diseases		Parkinson’s disease	Exacerbates neurodegeneration.	Promotes the intercellular spread of SNCA.	(56)
		Alzheimer’s disease	Improves cognitive function.	Restores lysophagy, recruits LC3, p62, and ubiquitin to damaged lysosomes, enhances lysosomal enzyme activity, reduces Aβ and p-MAPT accumulation.	(57)
		Age-related macular degeneration	Potential therapeutic target.	Mediates the release of harmful substances (e.g., Aβ, lipofuscin) via secretory autophagy.	(58)
Cardiovascular and cerebrovascular diseases		Myocardial ischemia/Reperfusion injury	Target for cardioprotective agents.	• Activates Keap1/Nrf2 antioxidant pathway. • Promotes NLRP3 inflammasome degradation, inhibiting pyroptosis.	(62, 63)
		Doxorubicin-induced cardiotoxicity.	Protective upregulation.	Degrades p-TAK1, inhibiting JNK/p38 inflammatory pathway and activating YAP/Nrf2 antioxidant pathway.	(59)
		Pathological cardiac hypertrophy.	Protective upregulation.	Degrades Src, activating Prdx1/Nrf2 antioxidant pathway.	(60)
		Stroke	Potential target for stroke treatment.	Downregulates KEAP1, activating NRF2/ARE antioxidant pathway, reducing apoptosis and ROS accumulation.	(64)
Inflammatory/Immune Diseases		Periodontitis	Prevent excessive inflammatory activation.	Binds proIL-1β and interacts with inflammasome components, enhancing IL-1β production	(28)
		Chronic obstructive pulmonary disease	Downregulated expression.	Mediates lysophagy to preserve lysosomal function.	(65)
Metabolic Diseases		Non-alcoholic steatohepatitis (NASH)	Reduces lipogenesis and promotes fatty acid oxidation.	Degrades p-TAK1, inhibiting JNK/p38 pathway.	(36)
Other diseases		Age-related sarcopenia	Alleviates oxidative stress.	Activates SIRT1/PI3K/AKT pathway, inhibiting E3 ligases associated with muscle atrophy.	(66)
		Senile osteoporosis	Promotes osteogenic differentiation.	• Activates p62-Keap1-Nrf2 antioxidant pathway. • Stabilizes Runx2 protein, enhancing ALP activity and mineralization.	(67)

## 6 Discussion

TRIM16 represents a non-canonical E3 ligase whose biological functions extend well beyond protein degradation. By participating in oxidative stress responses, secretory autophagy, lysophagy, immune modulation, and cell cycle regulation, TRIM16 contributes to the coordination of cellular homeostasis and stress adaptation. A defining feature of TRIM16 is its strong context dependency. It exerts tumor-suppressive effects in cancers such as neuroblastoma, colorectal cancer, and lung carcinoma, while promoting tumor progression in pancreatic cancer and subsets of hepatocellular carcinoma, underscoring the complexity of its regulatory networks. Beyond oncology, TRIM16 also displays protective roles in neurodegenerative, cardiovascular, metabolic, and inflammatory diseases, highlighting its broad pathophysiological relevance (**Figure 2**).

### 6.1 Clinical implications and therapeutic potential

The involvement of TRIM16 in diverse human diseases, supported by a growing body of preclinical evidence, highlights its potential value as a therapeutic target and biomarker, though its clinical translation requires careful context-specific evaluation. In oncology, TRIM16 functions as a context-dependent “double-edged sword.” In most solid tumors, including neuroblastoma, colorectal cancer, and non-small cell lung cancer, TRIM16 suppresses tumor progression by inducing apoptosis, inhibiting EMT, and reversing therapy resistance (7, 12, 36, 42, 47). In contrast, in pancreatic cancer and certain hepatocellular carcinomas, TRIM16 has been associated with enhanced tumor cell survival and metastasis (50, 52). Accordingly, therapeutic strategies targeting TRIM16 must be highly tailored based on robust tumor and molecular subtyping. Preclinical studies suggest that activating TRIM16 function—for example, using compounds such as sanggenon L in glioblastoma (49)—or restoring its expression, such as by targeting the circPTK2/miR-942 axis in non-small cell lung cancer (36), could be explored as a strategy in tumors where TRIM16 is tumor suppressive. Conversely, inhibition of TRIM16, as suggested by studies using delphinidin, may be required in cancers in which TRIM16 supports oncogenic signaling (51). The impact of TRIM16 on cancer goes far beyond its conventional signaling function; it delves into the core area of cancer genetics. In short, TRIM16 is influenced by germline polymorphisms, such as the rs2074890 mutation site present in hepatocellular carcinoma (41). There are also somatic epigenetic silencing phenomena, such as the occurrence of CRABP2/EZH2/DNMT1 in ovarian cancer, and a widespread downregulation phenomenon in many solid tumors, usually associated with advanced stages and poor prognosis (34). These genetic and epigenetic alterations make TRIM16 not only a downstream effector but also a direct genetic target or regulator in the tumor development process. This gene-oriented approach will be crucial for classifying patients and formulating targeted strategies that take into account the TRIM16 status in the individual tumor’s genetic structure.

Beyond cancer, preclinical studies suggest TRIM16 holds translational promise in chronic and age-related diseases. In neurodegenerative disorders such as AD, preclinical models indicate that TRIM16 overexpression restores lysosomal function and facilitates clearance of toxic protein aggregates (57). In cardiovascular and cerebrovascular diseases, TRIM16 limits tissue injury through antioxidant, anti-inflammatory, and anti-apoptotic mechanisms (59, 60, 62, 63). In chronic inflammatory conditions, including chronic obstructive pulmonary disease and periodontitis, enhancing TRIM16-mediated lysophagy and restraining inflammasome overactivation may reduce tissue damage (28, 65). Notably, TRIM16 is emerging as a modulator of the immune microenvironment. Its ability to regulate IL-1β secretion and promote degradation of immunosuppressive proteins such as Galectin-3 in glioma suggests potential relevance for immunotherapy (10, 40). Moreover, the association between TRIM16 expression and favorable responses to atezolizumab–bevacizumab in hepatocellular carcinoma patients warrants further validation of its potential as a predictive biomarker for immune checkpoint blockade (33). Together, these preclinical and early clinical findings highlight TRIM16 as a compelling molecule for further translational research, including the exploration of combinatorial therapeutic strategies.

### 6.2 Future perspectives

Despite substantial progress, the molecular determinants governing the context-specific functions of TRIM16 remain incompletely understood. Future studies should prioritize systematic dissection of TRIM16 interactomes and ubiquitinomes under defined pathological conditions, such as specific cancer subtypes or acute versus chronic inflammatory states, to clarify how its functional switch is regulated.

A critical gap lies in translating mechanistic insights into clinical practice. While numerous *in vitro* and preclinical studies delineate TRIM16’s roles, robust clinical data and prospective trial evidence linking TRIM16 status to patient outcomes or treatment responses are largely lacking. Future research must bridge this gap by conducting well-designed clinical cohort studies. These efforts should aim to validate TRIM16 expression levels, genetic variants (e.g., rs2074890), or epigenetic modifications as prognostic or predictive biomarkers for response to conventional therapies (chemotherapy, radiotherapy) and novel immunotherapies. Establishing such correlations is essential for patient stratification and personalized treatment planning. Future work should aim to systematically catalog TRIM16 genetic alterations across cancer types and link them to molecular subtypes, therapeutic responses, and patient outcomes, leveraging resources like cancer genome databases.

A major translational challenge lies in the lack of selective TRIM16 modulators. High-throughput screening and structure-based drug design targeting its B-box or SPRY domains may facilitate the development of compounds that selectively modulate TRIM16 activity or protein–protein interactions. In parallel, elucidating tissue- and cell-type specificity—including expression patterns, post-translational modifications, and availability of binding partners—will be essential.

Finally, given its involvement in tumor immune regulation and therapy resistance (33, 36, 40, 49), preclinical evaluation of TRIM16-based combination strategies with chemotherapy, radiotherapy, or immunotherapy is warranted. For diseases in which restoration of TRIM16 activity is desirable, such as neurodegenerative disorders (57), advanced delivery approaches including gene therapy, cell-based strategies, or nanotechnology-mediated delivery of TRIM16 mRNA or protein may further facilitate clinical translation.
